# Synthesis and Optical Properties of CdSeTe/CdZnS/ZnS Core/Shell Nanorods

**DOI:** 10.3390/nano14110989

**Published:** 2024-06-06

**Authors:** Geyu Jin, Yicheng Zeng, Xiao Liu, Qingya Wang, Jing Wei, Fangze Liu, Hongbo Li

**Affiliations:** 1 Beijing Key Laboratory of Construction-Tailorable Advanced Functional Materials and Green Applications, Experimental Center of Advanced Materials, School of Materials Science and Engineering, Beijing Institute of Technology, Beijing 100081, China; jingeyu1998@outlook.com (G.J.); yicheng_1026@163.com (Y.Z.); 3120221066@bit.edu.cn (X.L.); weijing@bit.edu.cn (J.W.); 2 Advanced Research Institute of Multidisciplinary Sciences, Beijing Institute of Technology, Zhuhai 519088, China; qingya6789@163.com (Q.W.); fliu@bit.edu.cn (F.L.); 3 Advanced Research Institute of Multidisciplinary Sciences, Beijing Institute of Technology, Beijing 100081, China

**Keywords:** CdSeTe/CdZnS/ZnS, core/shell, deep-red, nanorods, polarized emission

## Abstract

Semiconductor nanorods (NRs) have great potential in optoelectronic devices for their unique linearly polarized luminescence which can break the external quantum efficiency limit of light-emitting diodes (LEDs) based on spherical quantum dots. Significant progress has been made for developing red, green, and blue light-emitting NRs. However, the synthesis of NRs emitting in the deep red region, which can be used for accurate red LED displays and promoting plant growth, is currently less explored. Here, we report the synthesis of deep red CdSeTe/CdZnS/ZnS dot-in-rod core/shell NRs via a seeded growth method, where the doping of Te in the CdSe core can extend the NR emission to the deep red region. The rod-shaped CdZnS shell is grown over CdSeTe seeds. By growing a ZnS passivation shell, the CdSeTe/CdZnS/ZnS NRs exhibit a photoluminescence emission peak at 670 nm, a full width at a half maximum of 61 nm and a photoluminescence quantum yield of 45%. The development of deep red NRs can greatly extend the applications of anisotropic nanocrystals.

## 1. Introduction

Semiconductor nanocrystals (NCs) are considered as promising materials in the fields of light-emitting diodes (LED) [1,2,3], photovoltaics [4,5], sensors [6,7], photocatalysis [8,9,10], and photodetectors [11,12] due to their tunable band gaps, narrow emission bandwidth, and high photoluminescence quantum yield (PLQY) [13,14,15,16]. Isotropic spherical quantum dots (QDs) are the earliest discovered and most studied NCs, and their applications in the field of display have been widely studied. However, since the dipole moments in spherical QDs are randomly oriented, the theoretical maximum out-coupling efficiency (~20%) significantly limits the external quantum efficiency (EQE) of QLEDs [17,18]. On the other hand, the use of anisotropic nanocrystals including nanorods (NRs) and nanoplatelets (NPLs) as the light-emitting layer in QLEDs is expected to break the out-coupling efficiency limit and further improve the EQE of QLEDs [19].

Compared to QDs, nanorods (NRs) exhibit linearly polarized emission along their long axis [20,21]. NRs possess unique advantages that render them more promising than QDs in the field of lighting and displays owing to their larger Stokes shift, high absorption cross-section, and large light-emitting dipole spacing. Many reports have focused on optimizing the synthesis methods of NRs by using the core–shell structure. The core–shell structure can be broadly defined as a composite structure comprising an inner core and an outer shell, constructed from different material, and it is widely applied in various materials such as nanoparticles and microparticles to achieve superior chemical and physical properties [22,23,24]. Modeling and computational methods based on density functional theory have been used to guide the synthesis of core–shell structures [25,26]. Multi-shell structures with alloy transition shells have been shown to be effective in mitigating carrier delocalization and improving radiative recombinations [18,27,28]. In recent years, such core–shell structures have been increasingly explored in NRs [29,30]. Recently, Zeng et al. reported CdSe/CdZnS/ZnS NRs and CdSe/CdZnS/CdZnS/ZnS NRs with carefully designed multilayer shell structures, achieving the highest EQE of red NR-LEDs to date [31,32,33]. However, reports on the tunability of the emission wavelength of NRs are not as comprehensive as those on QDs. In addition to the most studied red NRs, Srivastava et al. achieved green and blue emitting NRs by diffusing Zn into the core to replace part of the Cd [29]. In addition, Manna et al. reported the conversion of CdSe/CdS NRs into ZnSe/ZnS NRs by cation exchange reaction, and the obtained NRs emitting in the blue-UV band [34]. Compared with green and blue NRs, reports on deep-red NRs (>650 nm) [35] are limited. Deep-red light presents unique application prospects across various fields. For example, the 670 nm deep-red light can aid in revitalizing the mitochondrial function among elderly individuals, thereby assisting in the restoration of color vision [36]. Deep-red light is the main absorption band for plant photosynthesis, and is commonly utilized to stimulate plant growth in greenhouses [37]. Moreover, in display applications, deep-red light can broaden the color gamut and deliver enhanced color rendering effects [38]. Therefore, there is a compelling demand to investigate the synthesis of deep-red NRs. However, due to the difficulty in reaching the deep-red region solely through modulation of the CdSe core size [39] and the lack of available materials that have an anisotropic growth of the wurtzite crystal structure, the synthesis of deep-red NRs remains to be explored [40,41].

Doping can achieve a wide-range tuning of the optical properties of nanocrystals without altering the structure of the host crystal [42,43,44]. Various dopants can induce a red shift in the luminescent emission of II-VI nanocrystals [45,46]. For example, when a small amount of Te atoms are doped in CdSe QDs, the emission peaks will be red-shifted compared to CdSe QDs of the same size due to the introduction of Te impurity energy levels near the CdSe valence band top [47,48]. Zhang et al. indicated that CdTe and CdSe QDs have different thermal sensitivities, and the temperature-dependent PL shift of CdSe_x_Te_1−x_ QDs can be controlled by increasing the content of Te, which is of great significance for reducing the influence of temperature on QLED devices [49]. While prior studies have reported on the doping of Te into CdSe/CdS nanorods (NRs), the luminescence performance of these deep-red NRs needs further improvement for practical applications [50,51]. Therefore, designing and optimizing the synthesis of core-shell deep-red NRs is of great importance for the development of high-performance deep-red light-emitting devices.

In this work, we synthesized deep-red CdSeTe/CdZnS/ZnS “dot-in-rod” core/shell NRs by doping a small amount of Te into the CdSe core. An alloyed CdZnS shell was designed to achieve better electron confinement and less lattice mismatch. Therefore, after the coating of the rod-shaped alloyed transition CdZnS shell and the ZnS outer shell, the final NRs exhibit an emission peak at 670 nm with a full width at half-maximum (FWHM) of 61 nm and a PLQY of 45%.

## 2. Materials and Methods

### 2.1. Materials

Cadmium oxide (CdO, 99.99%), trioctylphosphine oxide (TOPO, 99%), trioctylphosphine (TOP, 97%), selenium (Se, 99.99%), tellurium (Te, 99.99%), sulfur (S, 99%), and oleic acid (OA, 90%), were purchased from Sigma-Aldrich (St. Louis, MO, USA). 1-octadecene (ODE, 90%), hexylphosphonic acid (HPA, 98%), octadecylphosphonic acid (ODPA, 98%), and zinc acetate (Zn(ac)_2_, 99.99%) were purchased from Aladdin. All reagents were used without further purification.

### 2.2. Preparation of Precursors

Zn-OA (0.5 M): Zn(ac)_2_ (1.8348 g), ODE (10 mL), and OA (10 mL) were loaded into a 50 mL flask and dried for 1 h under vacuum at 120 °C. Then, the temperature was increased to 160 °C under an N2 atmosphere until the Zn(ac)_2_ was completely dissolved.

Se/Te-TOP (1.72 M): 0.74 mmol of a mixture of Se and Te was dissolved in 0.43 mL of TOP. For 5% Te doping, 0.055 g of Se and 0.005 g of Te were added.

S-TOP (2 M): 2 mmol of S powder was dissolved in 1 mL of TOP.

### 2.3. Synthesis of CdSeTe QDs

A hot-injection method was used to prepare CdSeTe QDs. TOPO (3.00 g), ODPA (0.280 g) and CdO (0.060 g) were placed in a three-neck round bottom flask and degassed at 150 °C for 1 h. Then, the solution was heated to 370 °C under an N_2_ atmosphere to dissolve the CdO until it turned clear and colorless; 1.8 mL of TOP was injected in the flask and the temperature was allowed to recover to 370 °C and 0.43 mL Se/Te-TOP solution was rapidly injected. The reaction was performed at 370 °C for 90 s, then the solution was cooled to room temperature. The crude solution was purified twice by dissolution in hexane and precipitation with methanol to remove unreacted impurities and precipitation. The obtained solid product was dissolved in TOP to form a clear solution with a 15 mg/mL concentration.

### 2.4. Synthesis of CdSeTe/CdS Core/Shell NRs

A seeded growth method was used to prepare CdSeTe/CdS NRs. CdO (0.0076 g), ODPA (0.041 g), TOPO (2.00 g), and HPA (0.011 g) were degassed at 130 °C for 90 min with vigorous stirring in a 50 mL round bottom flask, followed by three cycles of N_2_ purge. Then, the suspension was heated to 370 °C until it formed a transparent colorless solution. A total of 1.8 mL of TOP was injected, followed by raising the temperature to 375 °C. Once the solution reached 375 °C, a mixture of 0.8 mL TOP-S with 0.2 mL CdSeTe was quickly injected into the three-neck flask. The reaction was allowed to proceed for 8 min at 370 °C and then cooled to room temperature. The NRs were dispersed in hexane after two cycles of purification with hexane as a solvent and methanol as an antisolvent.

### 2.5. Synthesis of CdSeTe/CdZnS Core/Shell NRs

The synthesis of CdSeTe/CdZnS NRs was the same as that of CdSeTe/CdS NRs, except that 2 min after the injection of TOP-S and CdSeTe solution, 1 mL of Zn-OA was injected slowly at a rate of 0.2 mL/min, and the reaction was cooled to room temperature after 6 min. The obtained crude solution was purified with methanol and hexane. The NRs were finally dispersed in hexane.

### 2.6. Synthesis of CdSeTe/CdZnS/ZnS Core/Shell NRs

CdSeTe/CdZnS (8.5 mg dispersed in 2 mL hexane), Zn-OA (0.5 mL), and ODE (3 mL) were added to a 50 mL round bottom flask. The mixture was degassed in a vacuum at 120 °C for 1 h and then heated to 300 °C. One mL TOP-S was mixed with 2 mL ODE, and the mixture was injected slowly to the flask at a rate of 1 mL/min. The reaction was allowed to proceed within 1 min, followed by cooling down to room temperature. The CdSeTe/CdZnS/ZnS NRs were collected after two cycles of purification with hexane as a solvent and methanol as an antisolvent. The products were finally dispersed in hexane.

### 2.7. Characterizations

UV-vis absorption spectra were measured with a UV 2310-II spectrophotometer (Tianmei, Shanghai, China). PL spectra and PLQY values were collected with a Spectrofluorometer equipped with an integrating sphere (Edinburgh FS5, Edinburgh Instruments, Livingston, UK). X-ray diffraction (XRD) characterization was performed with a D8 Focus X-ray diffractometer (Bruker, Billerica, MA, USA). Transmission electron microscopy (TEM) images were acquired on a JEM-2100 electron microscope (JEOL Ltd., Tokyo, Japan) with an accelerating voltage of 200 kV. High-angle annular dark-field scanning transmission electron microscopy (HAADF-STEM) images and elemental maps were taken on an FEI Titan3 G2 60-300 TEM (FEI company, Hillsboro, OR, USA). X-ray photoelectron spectroscopy (XPS) was measured with an ULVAC-PHI QUANTERA-II SXM spectrometer (ULVAC, Chigasaki, Japan). The polarized PL measurements were carried out on a home-built fluorescence microscope with a laser diode (405 nm, 5 mW). A ½ waveplate was used to rotate the polarization angle of the PL from NRs. The PL signal then passed through a fixed linear polarizer and entered an Ocean optics spectrometer. The angular distribution of the PL emission was measured by rotating the ½ waveplate.

## 3. Results and Discussion

**Figure 1:**
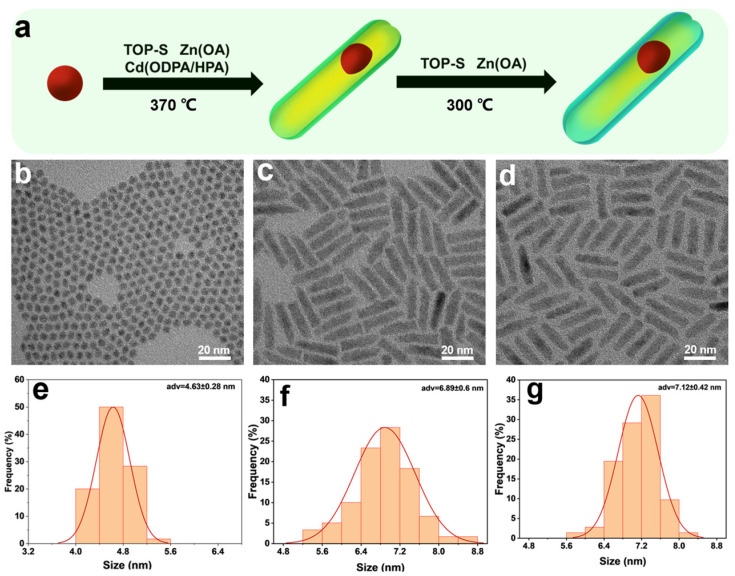
(**a**) Synthesis scheme of CdSeTe/S/ZnS NRs showing the growth of CdZnS transition shell and ZnS outer shell. (**b**–**d**) TEM images of (**b**) CdSeTe QDs, (**c**) CdSeTe/CdZnS NRs and (**d**) CdSeTe/CdZnS/ZnS NRs. Both CdSeTe seeds and NRs show uniform size distribution. The final CdSeTe/CdZnS/ZnS NRs exhibit highly uniform rod shape with smooth surface. (**e**) The diameter distribution of CdSeTe QDs showing an average diameter of 4.63 ± 0.28. Red line is the Gaussian fit to the histogram. The radial size distribution of (**f**) CdSeTe/CdZnS NRs and (**g**) CdSeTe/CdZnS/ZnS NRs showing an average diameter of 6.89 ± 0.60 nm and 7.12 ± 0.42 nm, respectively. The increased diameters demonstrate the growth of CdZnS and ZnS shells.

For the growth of CdSeTe quantum dots, we referred to the reported synthesis schemes for CdSe with a wurtzite structure [52,53]. The CdSe seeds were slightly doped with a small amount of Te by adjusting the anionic composition. The high reaction temperature of 370 °C is critical to maintaining the wurtzite structure of QDs, which is essential for the following rod-shaped shell growth [54]. In addition, the high temperature also helps to avoid Ostwald ripening and to obtain QDs with a uniform size [55,56]. Subsequently, the seeded growth method was used for the growth of a rod-shaped shell, as shown in Figure 1a. First, CdSeTe/CdZnS NRs were obtained by simultaneously injecting CdSeTe seeds and TOP-S as the S precursor into a three-necked flask containing the Cd precursor, followed by slowly dropwise adding the Zn-OA solution as the Zn precursor at 370 °C. Next, the TOP-S precursor was added dropwise to the solution containing CdSeTe/CdZnS NRs and the Zn-OA precursor to obtain the final CdSeTe/CdZnS/ZnS NRs.

The morphology and dimensions of the QDs are observed by transmission electron microscopy (TEM) as shown in Figure 1b–d. Size distribution statistics are shown in Figure 1e–g. The CdSeTe quantum dots as seeds had a narrow size distribution with an average diameter of 4.63 ± 0.28 nm. Nanorods were obtained after the CdZnS shell coating, with an average diameter of 6.89 ± 0.60 nm. The increased diameter and rod-shaped morphology provide preliminary evidence of the successful shell growth. After the ZnS shelling, the diameter of NRs slightly increased to 7.12 ± 0.42 nm, accompanied by an improvement in particle size uniformity and a change in the surface roughness, which proved the successful deposition of ZnS on the NR surface.

**Figure 2:**
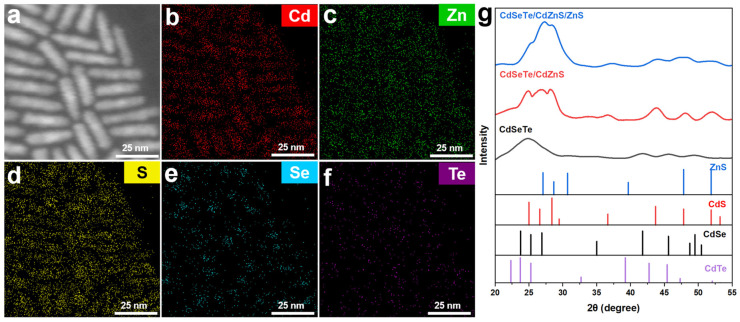
(**a**–**f**) The HAADF-STEM images of CdSeTe/S/ZnS NRs and the EDS mappings of Cd, Zn, S, Se and Te. The Se and Te atoms are mainly located in the inner core while the Zn and S atoms are distributed in the shells. Cd is distributed throughout the entire NRs, where the inner region had higher concentration and the outer region had lower concentration. These results prove the formation of a core–shell structure. (**g**) XRD patterns of CdSeTe QDs, CdSeTe/CdZnS NRs and CdSeTe/CdZnS/ZnS NRs. (CdSeTe: PDF#19-0193, CdSe: PDF#02-0330, CdS: PDF#01-0780, ZnS: PDF#0570). The CdSeTe QDs show a wurtzite structure. The XRD peaks gradually shift to higher angles due to the increased ZnS component.

Figure 2a–f shows the energy dispersive spectroscopy (EDS) mappings obtained from scanning transmission electron microscopy (STEM) for CdSeTe/CdZnS/ZnS NRs. Se is mainly located in the inner core, accompanied by a small amount of Te atoms. Cd is distributed throughout the entire NRs, where the inner region had higher concentration and its distribution shows a well-defined dot-in-rod structure. Zn and S are mainly distributed in the shells, proving the formation of a core–shell structure. The structures of CdSeTe QDs, CdSeTe/CdZnS NRs, and CdSeTe/CdZnS/ZnS NRs were further characterized via X-ray diffraction (XRD). As shown in Figure 2g, the synthesized CdSeTe QDs have a wurtzite structure. Since the deformation of the CdSe lattice is almost negligible due to the very small amount of Te doping, there is no obvious characteristic peak of CdTe, and only the broad characteristic peaks of CdSe are slightly shifted towards lower angles. The diffraction peaks of CdSeTe/CdZnS NRs are similar to those of CdS, which may be due to the low concentration of Zn. For CdSeTe/CdZnS/ZnS NRs, due to the increase in the ZnS component, the diffraction peaks slightly shifted to the high angles, and diffraction peaks at 27.08° and 47.83° showed increased intensity.

**Figure 3:**
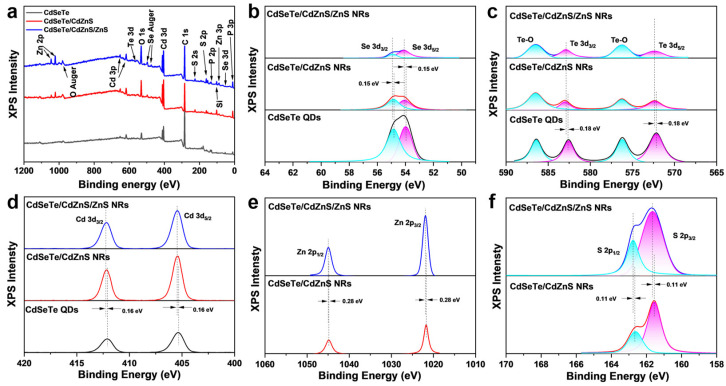
(**a**) The wide range XPS spectra of CdSeTe QDs, CdSeTe/CdZnS NRs and CdSeTe/CdZnS/ZnS NRs show the characteristic peaks of Cd, Se, and Te. The P peaks are from phosphorus-containing ligands of the nanocrystals, while Si, C, and O peaks are from the sample stage or air contamination. The high-resolution XPS spectra of (**b**) Se-3d, (**c**) Te-3d, (**d**) Cd-3d, (**e**) Zn-2p, and (**f**) S-2p with peak fitting showing the intensity evolution and peak shift after shell growth. The blue shift of Se-3d and Cd-3d peaks after shell growth proves the formation of the alloyed CdZnS shell. The shift of Zn-2p and S-2p peaks to higher energies demonstrates the growth of the ZnS shell.

In the X-ray photoelectron (XPS) spectra (Figure 3a), characteristic peaks of Cd, Se, and Te are shown in CdSeTe QDs, CdSeTe/CdZnS NRs and CdSeTe/CdZnS/ZnS NRs. Both the CdSeTe/CdZnS NRs and CdSeTe/CdZnS/ZnS NRs have additional characteristic peaks of Zn and S from the shell. The characteristic peaks of P are from phosphorus-containing ligands of the nanocrystals, while some characteristic peaks are assigned to impurity elements from the sample stage or air contamination such as Si, C and O. Figure 3b–d shows the high-resolution XPS spectra of Se-3d, Te-3d, Cd-3d, Zn-2p, and S-2p with peak fitting analysis. With the shell coating, the intensities of the Cd-3d peaks first increase and then decrease, while those of Se and Te gradually increase, and those of Se-3d and Te-3d continuously decrease. The trends of peak intensities clearly indicate the formation of the core–shell structure of NRs, which are also consistent with our previous report [31,32]. The fitting peaks of Se-3d, Te-3d, and Cd-3d shift to a higher binding energy after the shell growth, which proves the formation of the CdZnS shell. After growing a ZnS shell on the CdSeTe/CdZnS NRs, the fitting peaks of Zn-2p and S-2p also shift to a higher binding energy. Therefore, the high-resolution XPS data further confirmed the formation of the core–shell structure.

Compared with CdSe, a small amount of Te doping in CdSeTe QDs can produce impurity energy levels near the valence band top of CdSe, and the holes originally located in the valence band can relax to these impurity energy levels through non-radiative relaxation, resulting in red-shifted PL emission and a larger Stokes shift [48,50,51,57]. Though the increased amount of Te doping can achieve lower emission energy, it will have a detrimental effect on the subsequent growth of the shell. This may be due to the larger lattice distortion, as well as the lower stability of CdTe at high temperatures, which leads to the decomposition of the cores containing excess Te during shell coating. These effects will further lead to a large amount of self-nucleation of CdS, which is manifested by the impurity emission peaks at 500–600 nm in the PL spectrum (Figure S1). Therefore, it is necessary to control the doping amount of Te, which is found to be 5% of the total molar amount of the anion.

**Figure 4:**
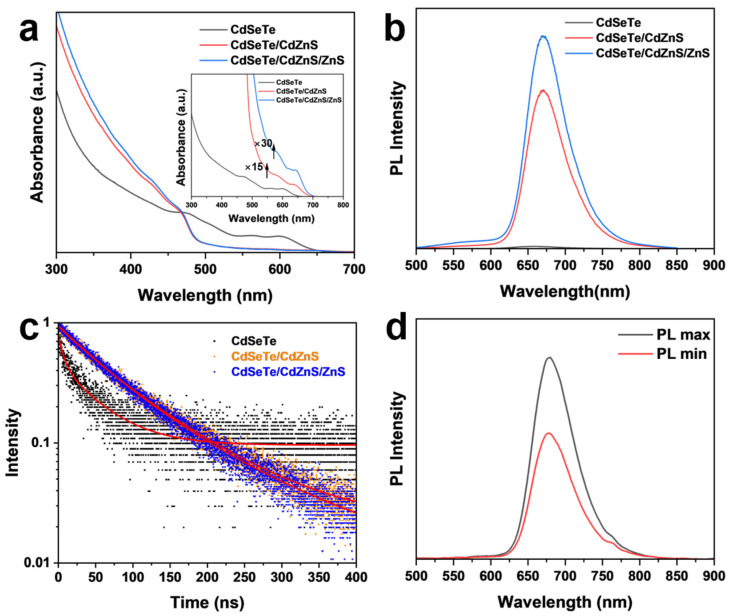
(**a**) The absorption spectra and (**b**) PL spectra (λ_ex_ = 450 nm) of CdSeTe QDs, CdSeTe/CdZnS NRs and CdSeTe/CdZnS/ZnS NRs, showing the evolution as the growth of shells. The significantly increased absorption between 300 nm and 450 nm demonstrates the successful growth of the wide bandgap shell. Inset: The same absorption spectra magnified by 15 (red line) or 30 (blue line) times to show the exciton peaks between 600 nm and 700 nm. (**c**) PL decay of CdSeTe QDs, CdSe/CdZnS NRs and CdSe/CdZnS/ZnS NRs, with the biexponential fitting showing as a red line. The PL lifetime increases from 50 nm of CdSeTe QDs to 90 nm of CdSeTe/CdZnS NRs and 100 nm of CdSeTe/CdZnS/ZnS NRs, proving the defect passivating effect of the shells. (**d**) Linearly polarized PL spectra of the CdSeTe/CdZnS/ZnS NRs solution were recorded at the minimum and maximum intensities, corresponding to the PL polarization parallel and perpendicular to the excitation light, respectively. The linearly polarized PL clearly demonstrates the anisotropic property of NRs.

The effects of shell coating on the absorption and PL emission spectra are shown in Figure 4a,b. CdSeTe QDs exhibit the first exciton absorption at 603 nm and PL emission at 656 nm with an FWHM of 72 nm. The inhomogeneous Te doping leads to a wide spread of the impurity energy levels [48,58], which leads to broadened PL emission and low PLQY (<5%). After the CdZnS coating, the absorption spectrum showed a characteristic increase of the shell below 500 nm, while the exciton absorption peaks of CdSeTe remained visible and redshifted to 640 nm (Figure 4a inset). The PL peak was redshifted to 670 nm with the FWHM narrowed to 61 nm, and the PLQY increased to 40%. After ZnS coating, the absorption in the 300–400 nm region was enhanced, and the PLQY of CdSeTe/CdZnS/ZnS NRs increased to 45%. These results showed that the alloyed shell coating led to an improved optical performance of NRs. The redshift of the spectrum is attributed to the delocalization of the electrons into the CdZnS shell. After the coating of the ZnS shell, the luminescence peak exhibited no further redshift, demonstrating the effective confinement of the electrons and holes [30,53]. However, prolonged growth or increased amount of the ZnS precursors can lead to excess ZnS on the NR surface and a decreased PL intensity (Figures S2 and S3) due to the increased interface defects caused by the lattice mismatch, which has been reported previously [59].

We further tested the time-resolved fluorescence (TRPL) spectra for CdSeTe QDs, CdSeTe/CdZnS, and CdSeTe/CdZnS/ZnS NRs, shown in Figure 4c. The fitting results with a double exponential decay are summarized in Table S1. CdSeTe QDs show an average PL lifetime of 45 ns due to the slower recombination of carriers captured by Te impurity levels compared to a band–edge recombination [51,60]. After the CdZnS coating, the PL lifetime increases to 90 ns, which is due to the delocalization of electrons into the shell and the passivation of the defect. The coating of the wide band gap ZnS further reduces the non-radiative recombination channels by passivating the surface defects, resulting in a further increase in the PL lifetime to 100 ns. The results of the TRPL agree well with the improving PLQY with a shell coating.

The anisotropic emission of NRs in the solution was resolved by measuring the linear polarization of the PL spectra parallel and perpendicular to the excitation light. The anisotropy value (*r*) is calculated by the following equation $r=\left(I_{∥}-I_{⊥}\right)/{(I}_{∥}+{2I}_{⊥})$, where $I_{∥}$ and $I_{⊥}$ are the PL intensities in the direction of the linear polarization parallel and perpendicular to that of the excitation light, respectively. A pure isotropic QD solution will show an *r* value of 0 while a perfectly linear polarized NR solution will show an r value of 0.4 [61]. For CdSeTe/CdZnS/ZnS NRs, Figure 4d shows a clear PL emission anisotropy with an *r* value of 0.221.

**Figure 5:**
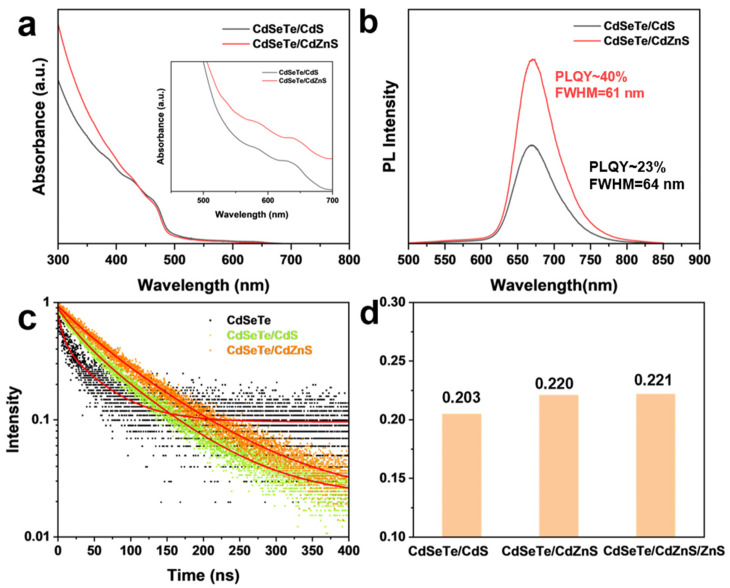
(**a**) The absorption spectra and (**b**) PL spectra (λ_ex_ = 450 nm) of CdSeTe/CdS NRs and CdSeTe/CdZnS NRs. Inset: Magnified absorption spectra showing the exciton absorption peaks. The CdSeTe/CdZnS NRs exhibit a higher PLQY of 40% than CdSeTe/CdS NRs (23%), proving the effective defect passivation of the alloyed shell compared with the conventional CdS shell. (**c**) PL decay of CdSeTe QDs, CdSeTe/CdS NRs and CdSeTe/CdZnS NRs, with the biexponential fitting showing as a red line. The CdSeTe/CdZnS NRs show a longer PL lifetime of 90 ns than that of CdSeTe/CdS NRs (75 ns) (**d**) The anisotropy value is increased from 0.203 of CdSeTe/CdS NRs to 0.220 of CdSeTe/CdZnS NRs and 0.221 of CdSeTe/CdZnS/ZnS NRs, which is due to the more uniform morphology of CdSeTe/CdZnS/ZnS NRs and the radial distribution of Zn in the CdZnS shell.

To further demonstrate the effect of the alloyed CdZnS shell, we also compared the optical properties of CdSeTe/CdZnS NRs with CdSeTe/CdS NRs, which were synthesized by the same procedures except for the injection of Zn-OA during the shell growth. As shown in Figure 5a, the CdSeTe/CdZnS NRs show higher absorption in the 300–400 nm region, which is characteristic of the growth of the CdZnS alloy shell. CdSeTe/CdS NRs have the same PL emission peak as the CdSeTe/CdZnS NRs, while the CdZnS shell results in a higher PL intensity (PLQY from ~23% to ~40%) and narrower peak widths (FWHM from 64 nm to 61 nm) (Figure 5b), which can be attributed to the passivation of the defects by Zn as well as a more uniform shell growth in the radial direction, as we noticed that the addition of Zn-OA resulted in better uniformity of the diameter of CdSeTe/CdZnS NRs than that of CdSeTe/CdS NRs (Figure S4). The high-quality shell coating improved the PL lifetime from 75 ns for CdSeTe/CdS NRs to 90 ns for CdSeTe/CdZnS, demonstrating the effective defect passivation (Figure 5c). These results correspond well with previously published reports [29,31,32]. The anisotropy value is increased from 0.203 of CdSeTe/CdS NRs to 0.221 of CdSeTe/CdZnS/ZnS NRs, which is due to the more uniform morphology of CdSeTe/CdZnS/ZnS NRs and the radial distribution of Zn in the CdZnS shell [33]. Note that the amount of Zn-OA must be precisely controlled, since high Zn content in the CdZnS shell will accumulate an excess strain to the CdSeTe core and increase the interface defect density, resulting in a lower PL intensity. The increased strain will also aggravate the type II band characteristics, and the delocalization of electrons into the shell will lead to an increase in the redshift of the PL peak and the PL lifetime (Figure S5).

## 4. Conclusions

In this work, we synthesized deep-red emitting CdSeTe/CdSeTe/CdZnS dot-in-rod core/shell NRs with a highly uniform morphology, which exhibit a PL emission at 670 nm with an FWHM of 61 nm and PLQY of 45%. The composition and structure of the core/shell NRs are confirmed by TEM, EDS mapping, XRD, and XPS. The light Te doping in the CdSeTe core redshifts the emission peak of CdSe NRs to the deep-red region, which is difficult for the pure CdSe to achieve. The rod-shaped CdZnS/ZnS alloyed shell improves the optical performance of NRs by effectively reducing the lattice mismatch and passivating the interfacial defects compared with a conventional CdS shell. As a result, the CdSeTe/CdSeTe/CdZnS NRs exhibit a higher PLQY, a longer PL lifetime and an improved linearly polarized PL with an *r* value of 0.221 in the solution. Our work extends the luminescence range of CdSe NRs into the deep red region, and the CdSeTe/CdSeTe/CdZnS NRs with a highly anisotropic emission can be a promising choice for the fabrication of high-efficiency deep-red luminescent NR devices.

## Data Availability

The original contributions presented in the study are included in the article/Supplementary Material, further inquiries can be directed to the corresponding author.

## Supplementary Materials

The following supporting information can be downloaded at: https://www.mdpi.com/article/10.3390/nano14110989/s1. Table S1. Summary of reaction conditions, PL peak wavelength and FWHM of CdSeTe QDs. Table S2. Biexponential fitting of CdSeTe QDs, CdSeTe/CdS NRs, CdSeTe/CdS NRs and CdSeTe/CdZnS/ZnS NRs PL lifetime results. Figure S1. The absorption spectra and PL spectra (λex = 450 nm) of CdSeTe QDs and CdSeTe/CdS NRs with the (a) 3%, (b) 5% and (c) 10% of Te containing in the anion mixture. Figure S2. The absorption spectra (a) and PL spectra (λex = 450 nm) (b) of CdSeTe/CdZnS/ZnS NRs during the ZnS coating with the extension of reaction time. Figure S3. The (a) absorption spectra and (b) PL spectra showing the effects of excessive Zn and S precursor on CdSeTe/CdZnS/ZnS NRs. (c) TEM image of the CdSeTe/CdZnS/ZnS NRs synthesized with excessive Zn and S precursor during the ZnS shelling procedure. Figure S4. The TEM image of (a) CdSeTe/CdS NRs and (b) CdSeTe/CdZnS NRs. Figure S5. The (a) absorption spectra and (b) PL spectra (λex = 450 nm) showing the effects of excessive Zn-OA on CdSeTe/CdZnS NRs. (c) TEM image of the CdSeTe/CdZnS NRs synthesized with excessive Zn-OA during the CdZnS shelling procedure. (d) PL decay of CdSeTe QDs, CdSe/CdS NRs and CdSe/CdZnS NRs. Inset: results of the biexponential fitting.
