# Supplementary material for: Synthesis and Optical Properties of CdSeTe/CdZnS/ZnS Core/Shell Nanorods
Source: Nanomaterials (Basel). 2024 Jun 6;14(11):989. doi: 10.3390/nano14110989 (PMC11173580; doi:10.3390/nano14110989)
Supplement: Supplementary file 1 [file nanomaterials-14-00989-s001.zip › nanomaterials-3017800-supplementary.pdf]

Supporting information

Synthesis and Optical Properties of CdSeTe/CdZnS/ZnS Core/Shell Nanorods

Geyu Jin [1,†], Yicheng Zeng [1,†], Xiao Liu [1], Qingya Wang [2,3], Jing Wei [1], Fangze Liu [2,3] and Hongbo Li [1,*]

[1] Beijing Key Laboratory of Construction-Tailorable Advanced Functional Materials and Green Applications, Experimental Center of Advanced Materials, School of Materials Science and Engineering, Beijing Institute of Technology, Beijing 100081, China; jingeyu1998@outlook.com (G.J.); yicheng_1026@163.com (Y.Z.); 3120221066@bit.edu.cn (X.L.); weijing@bit.edu.cn (J.W.)
[2] Advanced Research Institute of Multidisciplinary Sciences, Beijing Institute of Technology, Zhuhai 519088, China; qingya6789@163.com (Q.W.); fliu@bit.edu.cn (F.L.)
[3] Advanced Research Institute of Multidisciplinary Sciences, Beijing Institute of Technology, Beijing 100081, China
* Correspondence: hongbo.li@bit.edu.cn
† These authors have contributed equally to this work.

Table S1. Summary of reaction conditions, PL peak wavelength and FWHM of CdSeTe QDs.

Reaction temperature (°C)	Reaction time (s)	PL peak (nm)	FWHM (nm)
350	110	**656**	72
370	90	**656**	71
380	45	**656**	74

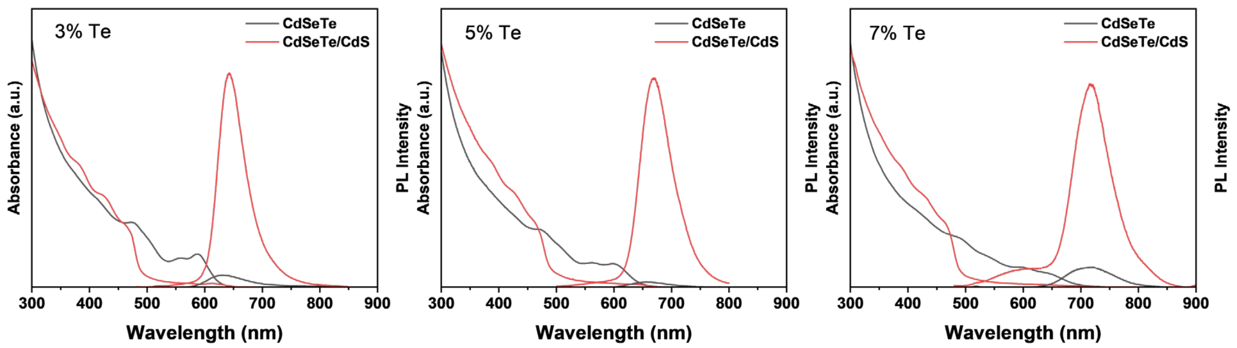

Figure S1. The absorption spectra and PL spectra ((λ_{ex}=450 nm) of CdSeTe QDs and CdSeTe/CdS NRs with the (a) 3%, (b) 5% and (c) 10% of Te containing in the anion mixture.

Table S2. Biexponential fitting of CdSeTe QDs, CdSeTe/CdS NRs, CdSeTe/CdS NRs and CdSeTe/CdZnS/ZnS NRs PL lifetime results.

	A1	t1	A2	t2	Tave(ns)
**CdSeTe**	0.33	6.89	0.36	50.05	45.31
**CdSeTe/CdS**	0.27	24.01	0.60	81.61	74.83
**CdSeTe/CdZnS**	0.26	40.77	0.65	98.59	90.27
**CdSeTe/CdZnS/ZnS**	0.58	57.77	0.35	131.59	100.26

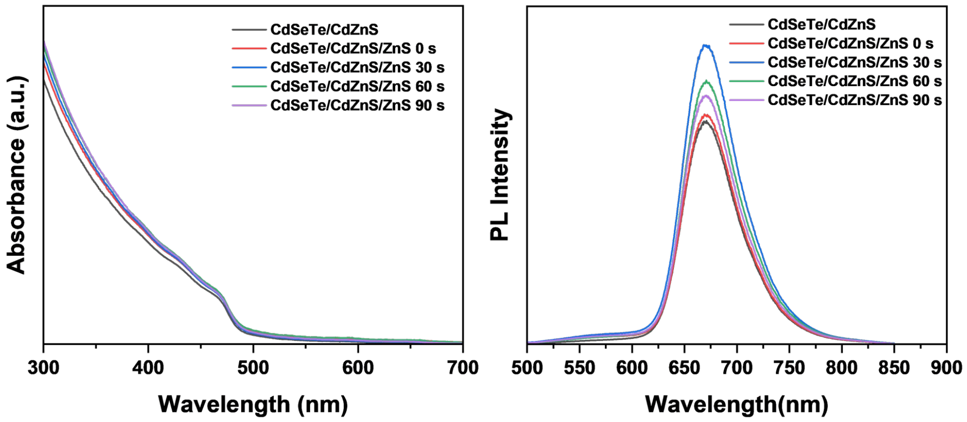

Figure S2. The absorption spectra (a) and PL spectra (λ_{ex}=450 nm) (b) of CdSeTe/CdZnS/ZnS NRs during the ZnS coating with the extension of reaction time.

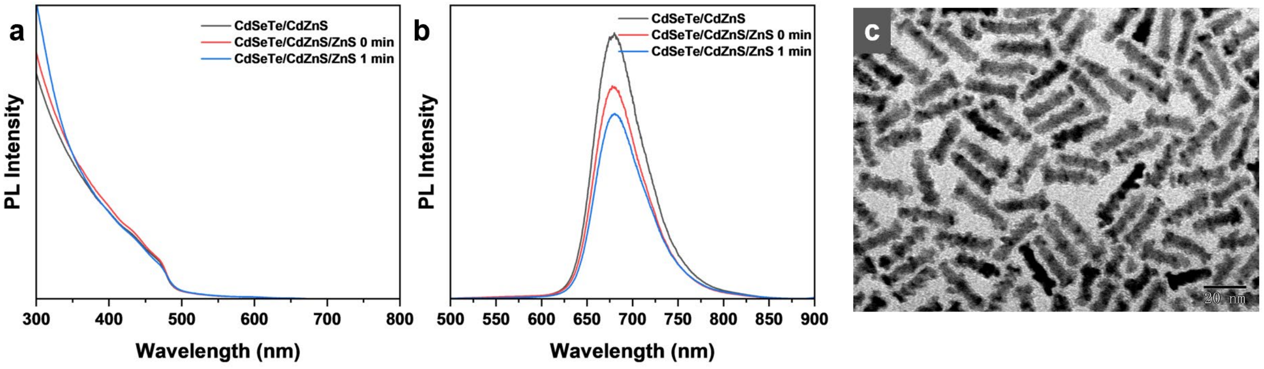

Figure S3. The (a) absorption spectra and (b) PL spectra showing the effects of excessive Zn and S precursor on CdSeTe/CdZnS/ZnS NRs. (c) TEM image of the CdSeTe/CdZnS/ZnS NRs synthesized with excessive Zn and S precursor during the ZnS shelling procedure.

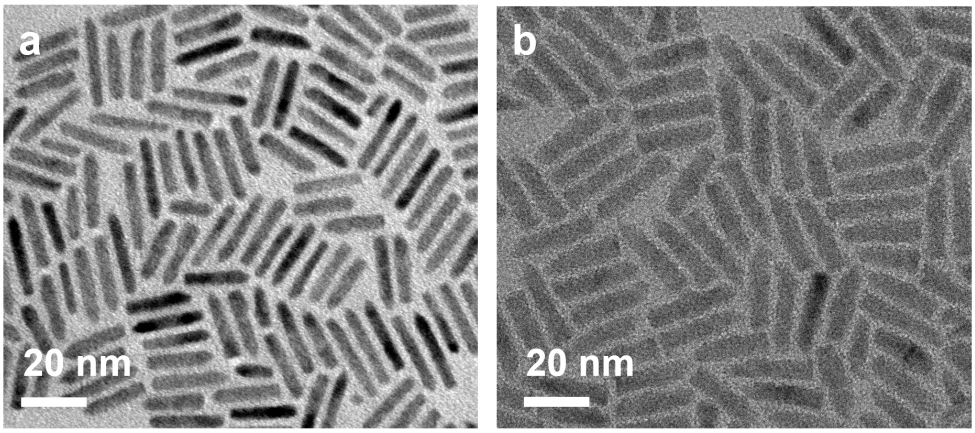

Figure S4. The TEM image of (a) CdSeTe/CdS NRs and (b) CdSeTe/CdZnS NRs.

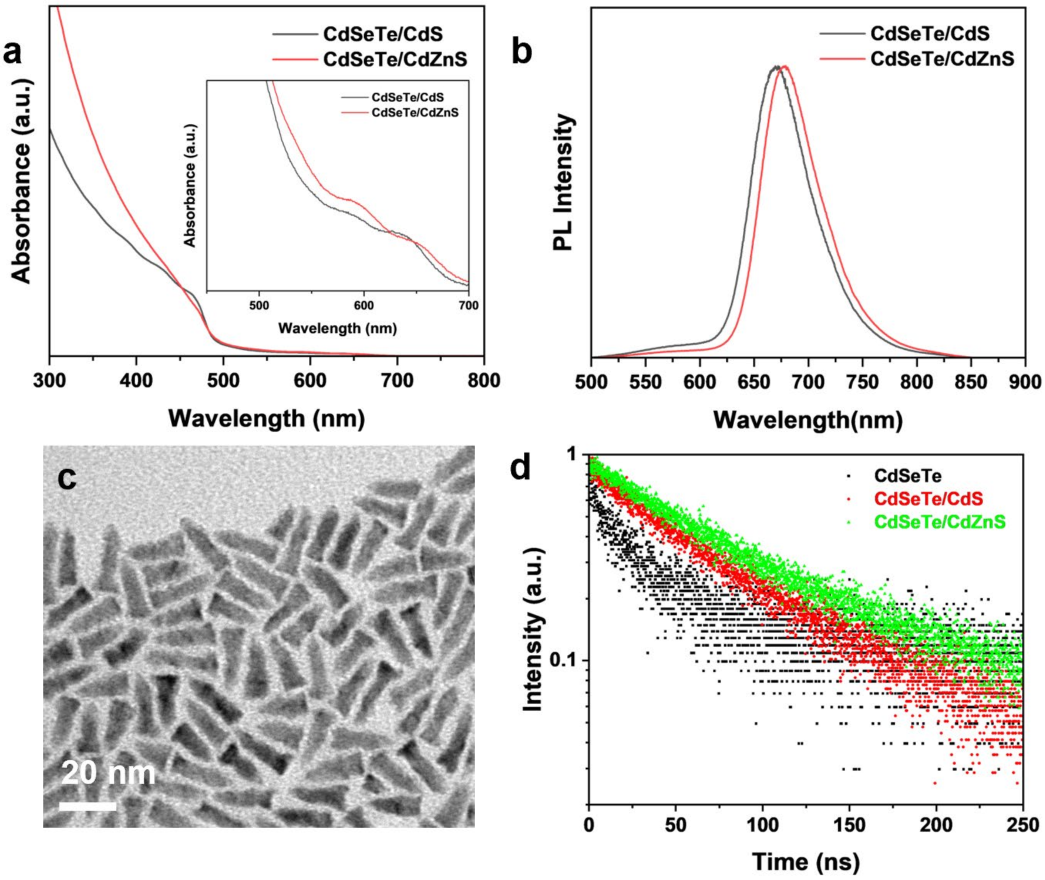

Figure S5. The (a) absorption spectra and (b) PL spectra (λ_{ex}=450 nm) showing the effects of excessive Zn-OA on CdSeTe/CdZnS NRs. (c) TEM image of the CdSeTe/CdZnS NRs synthesized with excessive Zn-OA during the CdZnS shelling procedure. (d) PL decay of CdSeTe QDs, CdSe/CdS NRs and CdSe/CdZnS NRs. Inset: results of the biexponential fitting.
